# Tobacco Use and Response to Immune Checkpoint Inhibitor Therapy in Non-Small Cell Lung Cancer

**DOI:** 10.3390/curroncol29090492

**Published:** 2022-08-30

**Authors:** Lucy K. Corke, Janice J. N. Li, Natasha B. Leighl, Lawson Eng

**Affiliations:** 1 Division of Medical Oncology and Hematology, Department of Medicine, Princess Margaret Cancer Centre, University Health Network Toronto, Toronto, ON M5G 2C1, Canada; 2 Division of Medical Oncology, Department of Medicine, University of Toronto, Toronto, ON M5S 1A8, Canada

**Keywords:** tobacco use, immune checkpoint inhibitors, lung cancer, clinical trials

## Abstract

Tobacco is a known risk factor for lung cancer, and continued tobacco use is associated with poorer outcomes across multiple lung cancer treatment modalities including surgery, chemotherapy and radiation therapy. Less is known about the association of tobacco use and outcomes with immune checkpoint inhibitors (ICIs), which are becoming an important part of the treatment landscape in lung cancer, both in metastatic and curative settings. We reviewed the literature on the association of tobacco and tumor biology as it relates to immunotherapy. We also reviewed the association of tobacco use on outcomes among phase III randomized clinical trials involving ICIs in non-small cell lung cancer (NSCLC). We identified that patients with a smoking history may have a greater benefit with ICI treatment compared to never smokers in both treatment-naïve (HR 0.82, 95% CI 0.69–0.97, vs. HR 1.06, 95% CI 0.81–1.38) and pre-treated (HR 0.79, 95% CI 0.70–0.90 vs. 1.03, 95% CI 0.74–1.43) settings. In trials where smoking status was further defined, ex-smokers appear to demonstrate greater benefit with ICI therapy compared to current smokers (HR 0.78, 95% CI 0.59–1.01 vs. 0.91, 95% CI 0.72–1.14). We conclude by offering our perspective on future directions in this area of research, including implementation of standardized collection and analysis of tobacco use in clinical trials involving ICI therapy in lung cancer and other disease sites, and also evaluating how tobacco may affect toxicities related to ICI therapy. Based on our review, we believe that a patient’s history of tobacco smoking does have a role to play in guiding treatment decision making in patients with lung cancer.

## 1. Introduction

Lung cancer is the leading cause of cancer-related mortality, contributing to almost 1.8 million deaths worldwide in 2020 [1]. Non-small cell lung cancer (NSCLC) accounts for 80% of cases, and two thirds are diagnosed at an advanced stage. Five-year survival rates for early and advanced-stage NSCLC are 57% and 6%, respectively [2]. Tobacco smoking is a major preventable risk factor for lung cancer, accounting for over 87% of lung cancer deaths [3].

The development of targeted therapies and immune checkpoint inhibitors (ICIs) have drastically changed the treatment landscape for NSCLC, improving drug tolerance and treatment outcomes. Inhibitors against programmed cell death protein 1 (PD-1) block the interaction between PD-1 and its ligand (PD-L1), restoring T-cell function and anti-tumour activity [4]. Anti-PD-1 agents are currently used as monotherapy, in combination with chemotherapy or other checkpoint inhibitors, depending on the patient’s PD-L1 tumour expression [5].

Durable long-term survival benefit with ICIs occurs in approximately 20% of patients with advanced NSCLC [6]. Accurate biomarkers to predict ICI response among lung cancer patients is currently lacking. Beyond PD-L1 tumour proportion score (TPS), other biomarkers such as tumour mutation burden (TMB), specific tumour mutations in TP53 and KRAS genes, inflammatory signatures including gamma interferon signalling and tumour-infiltrating lymphocytes (TIL) density have also been explored to predict benefit from anti-PD-1 agents [7,8]. Interestingly, several studies and clinical experience have shown that patients that are current or former smokers appear to have improved outcomes with anti-PD-1 therapy compared to never smokers [9,10]. This has been observed irrespective of PD-L1 expression [11,12], and may be related to the etiology and biological differences between lung cancer in smokers and non-smokers.

In this review, we summarize data from clinical trials of ICIs in patients with non-small cell lung cancer by smoking status. We also discuss the potential mechanisms through which ICIs may exert greater effects in the ever smoker population, compared to those patients with lung cancer driver mutations. Finally, we discuss the clinical significance of such an association and whether existing evidence is strong enough to guide treatment decisions in clinical practice.

## 2. Tobacco Smoking and the Immune System

**Figure 1:**
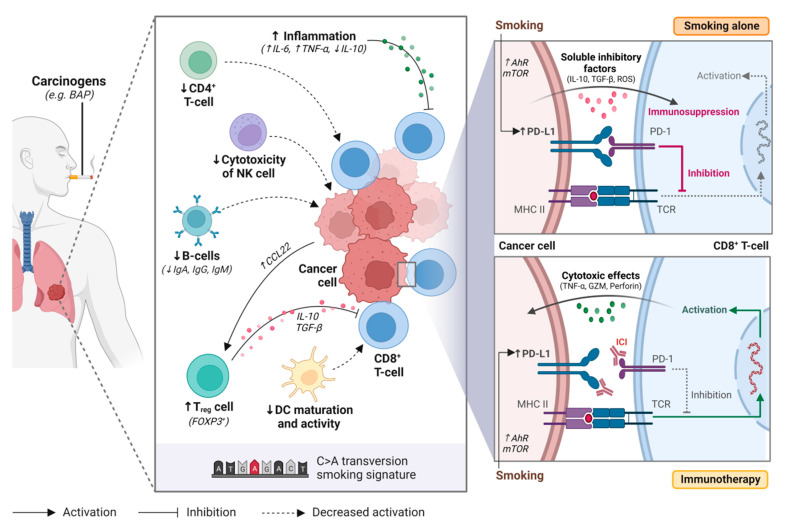
Tobacco smoking alters the immune and tumour microenvironment of lung cancer. Smoking may impair anti-tumour activity through over-activating the inflammatory immune response, upregulating regulatory T cell (Treg) function, decreasing natural killer (NK) cell cytotoxicity, downregulating B-cell activity and antibody production (i.e., IgA, IgG, IgM), and supressing dendritic cell (DC) maturation and activity. Smoking also increases programmed death-ligand 1 (PD-L1) expression on cancer cells through aryl hydrocarbon receptors (AhR)-related pathways or mTOR signaling, resulting in T-cell inactivation. Immune checkpoint inhibitors restore immune activation through disrupting the programmed death 1 (PD-1)/PD-L1 interaction. BAP, benzo[a]pyrene; NK cell, natural killer cell; Treg cell, regulatory T cell; DC, dendritic cell; Ahr, aryl hydrocarbon receptor; mTOR, mechanistic target of rapamycin; ICI, immune checkpoint inhibitor; PD-1, programmed cell death protein 1; PD-L1, programmed death-ligand 1; MHC II, major histocompatibility complex II; TCR, T-cell receptor. Figure created with Biorender.com.

A single cigarette contains over 7000 hazardous chemicals and 60 carcinogens, including polycyclic hydrocarbon carcinogens such as benzo[a]pyrene (BAP) and nicotine-derived nitrosoaminoketone [13,14]. Polycyclic hydrocarbons are responsible for the formation of bulky DNA adducts and their removal by nucleotide excision repair may result in DNA damage, tumorigenesis, and enhanced mutation burden [15,16,17]. This creates a distinct mutational signature in smokers (smoking signature, SS) characteristic of C>A transversions [18]. Tobacco smoking is also associated with the upregulation of PD-L1, which impairs the inflammatory response, allowing tumor cells to evade the immune system [19,20,21] (Figure 1).

In healthy individuals, the PD-1/PD-L1 axis dampens the immune system to protect the host against autoimmunity. However, tumor cells use this same mechanism to evade the immune system [19]. Tobacco smoking is associated with high tumor PD-L1 expression and has a dose-dependent effect [19,21]. While the exact mechanism responsible for this phenomenon remains unclear, it has been hypothesized that tobacco carcinogens such as BAP may be responsible for upregulating PD-L1 expression [19]. BAP activation is mediated by aryl hydrocarbon receptors (AhR), that detoxify xenobiotics and regulate immune cell function [22]. Wang et al. observed that silencing AhR in mice decreased PD-L1 expression and prolonged survival [19]. Furthermore, treatment with anti-PD-1 agents suppressed tumor formation and significantly enhanced T-cell infiltration. In lung cancer patient samples, they observed higher AhR and PD-L1 expression in smokers compared to never smokers. Patients with high AhR-expressing tumors had greater clinical benefit with pembrolizumab, including in multivariable analysis [19].

Xiao et al. have proposed that mTOR signaling is responsible for stimulating PD-L1 expression through IL-6 [20]. When inhibiting mTOR using rapamycin, a significant reduction in PD-L1 expression was observed in experimental models. The addition of IL-6 recombinant protein to tobacco-cultured cell lines increased PD-L1 expression and partially restored tobacco-stimulated PD-L1 expression in rapamycin-treated cell lines. Together, these findings suggest that tobacco carcinogens play a role in increasing PD-L1 expression, and that these mechanisms may improve response to anti-PD-1 agents.

### 2.1. Smoking and Immune Cells

Chemicals found within tobacco cigarettes can impede the development, effector function and cytokine production of the immune system and alter the tumor immune microenvironment [23,24,25]. Through increasing the production of pro-inflammatory cytokines and reducing anti-inflammatory cytokine production, smoking induces an inflamed tumor microenvironment that promotes tumorigenesis and leads to chronic stimulation of T-cells with subsequent exhaustion and activation-induced cell death [23,26,27,28].

CD8^+^ tumor-infiltrating lymphocytes (TILs) are crucial for eliciting a direct cell-mediated antitumor immune response [29]. A combination of high CD8^+^ and low regulatory T-cell (T_reg_) infiltration is associated with prolonged survival in cancer patients [30,31]. High densities of tumor stromal infiltrating CD4^+^ and CD8^+^ T-cells have been significantly associated with smoking status in NSCLC [32] and other lung diseases [33]. However, other studies suggest no association between TIL density and smoking status [34,35]. Hiraoka et al. observed that simultaneously high densities of CD4^+^ and CD8^+^ T-cells have a synergistic effect and are associated with a favorable prognosis in lung cancer [36]. Despite the high abundance of CD8^+^ T-cells in the tumor stroma, they are functionally impaired and poorly responsive to T-cell activating stimuli [26,37]. This may be due to ineffective tumor-antigen presentation by dendritic cells (DC), reduced activity of CD4^+^ T-cells, and regulatory functions by T_reg_ cells [26].

The highly inflammatory immune response associated with smoking also favors the accumulation and activation of DC [38,39]. However, smoking may also suppress DC function and maturation [26]. In tumor supernatants, Sharma et al. demonstrated that DCs had reduced surface expression of major histocompatibility complex (MHC) class I and II and co-stimulatory molecules (e.g., CD80 and CD86) in vitro, reducing their capacity to present tumor-specific antigens for T-cell activation [40]. Similar findings were observed in mice [27,41] and human DC cell lines [42], where exposure to cigarette smoking extracts hindered DC ability to stimulate and activate antigen-specific T-cells. Thus, cigarette smoking appears to have a profound impact on DC function and subsequent immunosuppression.

CD4^+^ T-cells are important for the initiation and maintenance of CD8^+^ T-cell activity [43]. T_reg_ cells are a subset of CD4^+^ effector T-cells that maintain immunological homeostasis through immunosuppression [44]. Depletion of T_reg_ cells has previously been demonstrated to augment anti-tumor T-cell activity [45,46]. Smoking status is associated with a low frequency of CD4^+^ T-cells [47,48] and high frequency of T_reg_ cells [31]. Kinoshita et al. observed a high FOXP3/CD4 ratio in smokers with lung adenocarcinoma, where FOXP3 regulates T_reg_ development, and suggested that this was an unfavorable prognostic factor [47]. Likewise, Sato et al. showed significant association between lung adenocarcinomas with a smoking signature (C > A transversions) and FOXP3^+^ T-cells [49]. These studies demonstrate that tumors with smoking signatures were associated low expression of CD4^+^ T-cells and high levels of T_reg_ cells, leading to reduced tumor-infiltrating CD8^+^ T-cell activity and poor prognosis in NSCLC. T_reg_ cells can also act on CD8^+^ T-cells indirectly through suppressing DC function [50], preventing proper antigen presentation to T-cells, leading to immunosuppression.

### 2.2. Smoking, Tumor Mutational Burden (TMB) and the Genomic Landscape

There is mounting evidence that smoking status and smoking signatures in tissues are associated with high somatic mutation rates, neoantigen production, and higher TMB [49,51,52,53,54,55]. There is conflicting evidence as to whether or not this is a dose-dependent effect. In NSCLC patients, Nagahashi et al. showed that current smokers without oncogenic driver mutations had the highest TMB in their samples, (*p* < 0.01), as compared to former or never smokers [53]. No differences in TMB were observed based on duration or intensity of tobacco exposure or pack years (PY). In contrast, Wang et al. observed that a doubling of smoking PY in NSCLC patients was associated with 1.11-fold increase in TMB (*p* < 0.001). Doubling of the number of months since quitting smoking was associated with a 0.95-fold decrease in TMB (*p* < 0.001) [54]. In summary, smoking is strongly associated with TMB, which may be associated with improved treatment outcomes with anti-PD- therapy [55,56].

The NSCLC mutational landscape in smoking and never smoking patients differs considerably. Tobacco smokers often have a tumor genomic profile characteristic of high frequencies of C > A transversions and defective DNA mismatch repair [18,54]. Specific signatures are associated with smoking-induced lung cancer. Nik-Zainal et al. demonstrated that exposure to BAP could induce similar genomic signatures [57]. Never smokers, on the other, have distinct genomic profiles, characteristic of spontaneous deamination of 5-methylcytosine and ultraviolet-induced mutations, respectively [54]. NSCLC patients with a smoking history or tumors with smoking-related signatures had better response rates and survival outcomes with anti-PD-1 treatments than never smokers or those with tumors without smoking-related signatures [51,58].

## 3. Moving into Clinic–Anti-PD(L)1 Checkpoint Inhibitors, Smoking and Outcomes

We reviewed published registrational, phase 3 and randomized phase 2 clinical trials involving ICIs in NSCLC patients, where data was available to assess the impact of tobacco use on trial end points. We identified landmark clinical trials and subsequently also reviewed trials on clinicaltrials.gov and similar systematic reviews evaluating ICI in lung cancer to help with identifying relevant ICI trials in lung cancer. Preliminary data presented as abstracts at recent meetings were included where the results of the study were felt to be practice-changing. Among these studies, the following data were extracted from individual publications (or abstracts, if applicable): title, authors, year, sample size, inclusion/exclusion criteria (including PDL1 status, histological subtypes, line of therapy), treatment and comparator arms and intervention details, smoking status as well as sample size in each tobacco status group. Associations between smoking status and outcome data were recorded as HR along with their respective confidence intervals (CI). We noted whether studies were analyzed based on ever vs. never smoking status or ex-smoker vs. current smoker vs. never smoker status. Meta-analyses were conducted, and forest plots were created to help summarize and evaluate the overall prognostic associations of smoking status on ICI therapy outcomes based on the line of therapy when there were an adequate number of individual studies with similar reporting of tobacco status, line of therapy and outcomes to justify such an analysis. Only studies reporting HR for OS were included in the forest-plots. Data were analyzed using RevMan 5.4 (The Cochrane Collaboration, 2020) to summarize the data and compare across subgroups. Pooled estimates of HR were computed using generic inverse variance and a random-effects model [59,60]. All statistical tests were 2-sided, and statistical significance was defined as *p* < 0.05.

### 3.1. Previously Treated Advanced NSCLC

In the phase I KEYNOTE-001 trial, previously treated patients with advanced NSCLC who received pembrolizumab were found to have an estimated 5-year overall survival (OS) of 15.5% [6]. Subgroup analysis found that current or former smokers had a 5-year OS rate of 16.8% (95% CI 12.8 to 21.3) compared to never smokers at 12% (95% CI 6.9–18.6). The subsequent phase III KEYNOTE-010 trial which randomized previously treated patients with tumor PD-L1 expression ≥1% to pembrolizumab or docetaxel demonstrated durable survival benefit at the 5 year survival update with pembrolizumab, especially in patients with PD-L1 TPS ≥50% [61]. Subgroup analysis, however, showed that both groups benefitted similarly in terms of current/former smokers and never smokers (HR for OS 0.69, 95% CI 0.59–0.81, and 0.79, 95% CI 0.47–0.95, respectively) [61].

Pooled analysis of the phase III CheckMate-017 and CheckMate-057 trials comparing nivolumab to docetaxel in previously treated patients with squamous and non-squamous NSCLC, respectively, demonstrated a durable survival benefit with nivolumab regardless of tumor histology [62]. Median OS in the overall study population was 11.1 months for nivolumab compared to 8.1 months for docetaxel (HR 0.68, 95% CI 0.59–0.78) [62]. In patients with a smoking history, median OS was 10.7 months with nivolumab compared to 7.9 months with docetaxel (HR 0.63, CI not reported). Never smokers did not have significantly different outcomes with nivolumab, (median OS 12.8 months), compared to docetaxel (median OS 9.2 months, HR 0.99) [62].

The phase III OAK study compared PD-L1 inhibitor atezolizumab to docetaxel. In an updated analysis with 1225 patients, atezolizumab improved overall survival across histological and PD-L1 subgroups (median OS 13.3 versus 9.8 months, HR 0.80, 95% CI 0.70-0.92) [63]. Median OS for current/former smokers was 9.3 months with docetaxel, compared to 13.1 months with atezolizumab (HR 0.78, 95% CI 0.67–0.90). Never smokers were not observed to have the same difference in benefit although they had a better prognosis overall–median OS was 13.6 months in the docetaxel arm and 14.1 months with atezolizumab (HR 0.91, 95% CI 0.65–1.29) [63].

Finally, in the phase III JAVELIN Lung 200 trial, the PD-L1 inhibitor avelumab was compared to docetaxel in patients with previously treated PD-L1 positive advanced NSCLC. At 2 years of follow-up, overall survival was not significantly improved with avelumab except in patients with high PD-L1 tumor expression [64]. There was a non-significant trend toward improved survival in patients with a smoking history, with a median OS of 10.6 months in the avelumab arm and 8.6 months in the docetaxel arm (HR 0.82, 95% CI 0.67–1.02). Patients that were never smokers appeared to have a trend to worse outcome in the checkpoint inhibitor arm, with a median OS of 13.9 months with avelumab and 18.5 months with docetaxel (HR 1.29, 95% CI 0.78–2.12).

**Figure 2:**
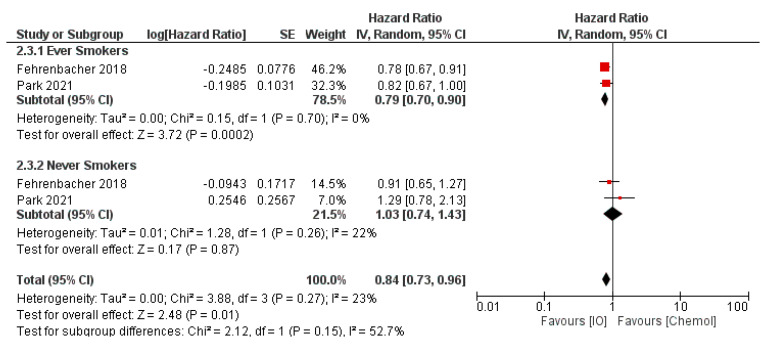
Pooled analysis of overall survival with second-line immunotherapy or chemotherapy compared by smoking status at study entry. Created with RevMan version 5.4, the Cochrane Collaboration, 2020 [63,64].

Thus, the impact of single agent anti-PD-1 or -PD-L1 therapy in advanced pretreated NSCLC appears to be greater in patients with a smoking history compared to those without compared to second-line chemotherapy (Figure 2).

### 3.2. Treatment Naïve Advanced NSCLC

#### 3.2.1. First Line Anti-PD(L)1 Monotherapy

Five year OS in the KEYNOTE-001 trial for all treatment naïve patients that received pembrolizumab was 23.3% [6]. Patients with a smoking history had an estimated 5-year OS of 26.4% (95% CI 17.6 to 36) although there were too few never smoking patients at 5 years of survival follow up to enable a comparison.

In KEYNOTE-024, treatment naïve advanced NSCLC patients with PD-L1 tumor proportion score (TPS) ≥ 50% were randomized to pembrolizumab or platinum-based chemotherapy and demonstrated a durable survival benefit with pembrolizumab at 5 years (median OS 26.3 months versus 13.4 months, HR 0.62, 95% CI 0.48–0.81) [65]. Greater benefit was seen in former smokers (HR 0.59, 95% CI 0.41–0.85), while current smokers (HR 0.81, 95% CI 0.41–1.60) and never smokers (HR 0.90, 95% CI 0.11–7.59) appeared to derive less benefit [66].

The subsequent KEYNOTE-042 study assessed pembrolizumab or platinum-based chemotherapy in treatment naïve advanced NSCLC patients with PD-L1 TPS ≥ 1%, and similarly found that pembrolizumab provided greater survival benefit, driven largely by those with TPS scores ≥50% [67]. Subgroup analysis of all patients (TPS ≥ 1%) demonstrated again that the largest benefit was in former smokers (HR 0.71, 95% CI 0.59–0.86). Current smokers (HR 0.95, 95% CI 0.70–1.29) and never smokers (HR 1, 95% CI 0.73–1.37) appeared to derive less benefit and this trend was seen regardless of TPS cut-off, i.e., 1% or ≥50% [18].

A similar trend was seen in the phase III IMpower 110 study, which demonstrated a survival benefit of atezolizumab monotherapy compared to platinum doublet chemotherapy for patients with PD-L1 tumor cell expression ≥50% [68]. Within this population, current and former smokers appeared to benefit more than never smokers although this was not significantly different (current smokers HR 0.61, 95% CI 0.30–1.24; former smokers HR 0.75, 95% CI 0.47–1.18; never smokers HR 1.98, 95% CI 0.77–5.08, respectively) [69].

The phase III MYSTIC and CHECKMATE-026 studies of durvalumab and nivolumab, respectively, versus platinum chemotherapy in patients with PD-L1 positive (tumor cell 1% or more) NSCLC both failed to meet their primary endpoints of improved overall survival [56,58]. In MYSTIC, patients with PD-L1 tumor cell expression ≥25% treated with durvalumab had a statistically non-significant reduction in the risk of death [56]. There was no difference seen in OS by smoking status. Similarly in CHECKMATE-026, nivolumab did not significantly improve PFS for patients with PD-L1 tumor cell expression ≥5% [58]. In subgroup analysis, there was no difference in survival by smoking status, although never smokers appeared to have a trend to worse progression-free survival (PFS) compared to former or current smokers.

**Figure 3:**
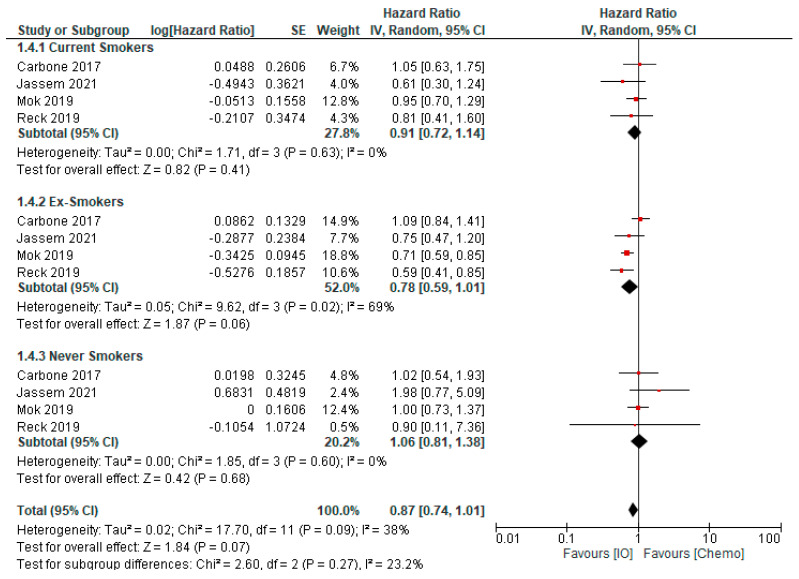
Pooled analysis of overall survival in treatment naïve patients with advanced NSCLC treated with first-line immunotherapy or chemotherapy, compared by smoking status at study entry. Created with RevMan version 5.4, the Cochrane Collaboration, 2020 [58,66,67,69].

**Figure 4:**
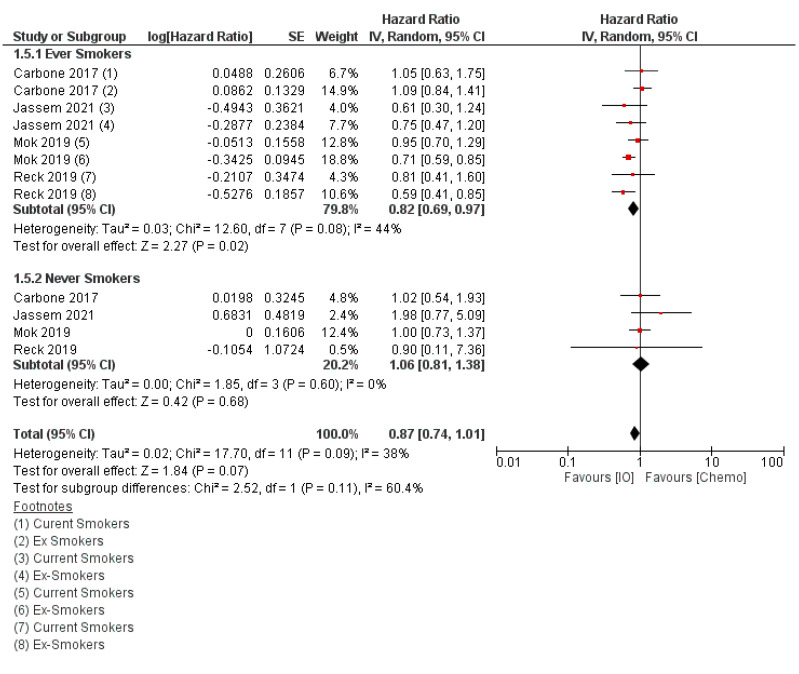
Overall survival with first-line immunotherapy or chemotherapy by smoking status at study entry. “Ever” smokers includes both current and former smokers. Created with RevMan version 5.4, the Cochrane Collaboration, 2020 [58,66,67,69].

We have combined these results across studies in Figure 3, illustrating that never smokers do not appear to derive significant survival benefit from PD-(L)1 inhibitor monotherapy. In contrast, current smokers have a trend to improved survival and a more pronounced effect for former smokers. When we combine current and former smokers, (Figure 4), ever smokers appear to derive significant survival benefit from anti-PD-(L)1 monotherapy compared to chemotherapy, while never smokers do not.

#### 3.2.2. Anti-PD(L)1 and Chemotherapy Combinations

KEYNOTE-189 demonstrated significantly longer overall survival with the addition of pembrolizumab to platinum-pemetrexed chemotherapy in patients with treatment naïve, advanced non-squamous NSCLC, regardless of PD-L1 TPS [70]. Median OS in all patients was 22 months in the combination group compared to 10.6 months in the chemotherapy arm (HR 0.56, 95% CI 0.46–0.69) [71]. A significant benefit was seen with combination therapy in both ever smokers, (HR 0.54, 95% CI 0.41–0.71) and never smokers (HR 0.23, 95% CI 0.10–0.54), albeit with smaller numbers [70]. This has clinical relevance given the poor outcomes with anti-PD-(L)1 monotherapy in never smokers in the studies described earlier. While KEYNOTE-407 demonstrated improved survival with the addition of pembrolizumab to platinum-taxane chemotherapy in treatment naïve patients with advanced squamous lung carcinoma, subgroup analysis by smoking status was not performed as over 90% of patients had a smoking history [72].

Atezolizumab has also improved outcomes when used in combination with chemotherapy in both squamous and non-squamous NSCLC [73,74]. Subgroup analyses from IMpower 130 and IMpower 131 both showed a trend to improved survival with combination therapy in never and ever smokers, but this was not significant. IMpower150 randomized treatment naïve patients with advanced non-squamous NSCLC to receive either bevacizumab, paclitaxel and carboplatin plus atezolizumab or placebo, or paclitaxel, carboplatin and atezolizumab [75]. While atezolizumab plus chemotherapy did not improve survival in the overall population compared to bevacizumab plus chemotherapy, ever smokers derived significant benefit in the comparison of the two regimens (HR 0.83, 95% CI 0.69–0.99; HR 0.92, 95% CI 0.64–1.32) [76]. The four drug combination of atezolizumab, bevacizumab and chemotherapy resulted in significant improvement in overall survival compared to bevacizumab plus chemotherapy alone [76]. This benefit was seen across key subgroups including never smokers and those with *EGFR* and *ALK* driven lung cancer. Patients who were current or former smokers had a median survival of 19.0 months with atezolizumab plus chemotherapy, and 14.1 months with bevacizumab plus chemotherapy (HR 0.82, 95% CI 0.69–0.98), suggesting that anti-PD-(L)1 therapy may be more effective in patients with tobacco exposure.

Cemiplimab in combination with chemotherapy improved survival compared to placebo-chemotherapy in EMPOWER-Lung 3 (median OS 21.9 versus 13 months, HR 0.71, 95% CI 0.53–0.93) [77]. Exploratory analysis by smoking status demonstrated that ever smokers derived much greater benefit (HR 0.61, 95% CI 0.46–0.82) than never smokers, who appeared not to benefit at all with addition of ICI (HR 1.28, 95% CI 0.53–3.08).

Newer PD-1 inhibitors have also been evaluated in combination with chemotherapy.

Camrelizumab and sintilimab have also demonstrated survival benefit in combination with chemotherapy in patients with advanced squamous and non-squamous NSCLC, respectively, in large studies conducted in Asia. In the CAMEL-SQ study, the addition of camrelizumab to chemotherapy in treatment naïve patients with advanced squamous lung carcinoma improved overall survival [78]. In patients with a heavy smoking history (defined as greater than 400 cigarette-years), chemotherapy plus camrelizumab led to a significant survival benefit (HR 0.52, 95% CI 0.37–0.73) [78]. There were limited numbers of patients that were light or never smokers, thus the impact of combination treatment in this population is less clear (HR 0.92, 95% CI 0.31–2.73). In ORIENT-11, sintilimab in combination with platinum-pemetrexed also showed a significant survival benefit for patients with advanced non-squamous NSCLC and a smoking history (HR 0.54, 95% CI 0.389–0.756). However, the benefit was not statistically significant in the subgroup that had never smoked (HR 0.753, 95% CI 0.449–1.263) [79].

#### 3.2.3. Anti-PD(L)1 and Anti-CTLA-4 Combinations, with or without Chemotherapy

CheckMate227 demonstrated improved survival with the combination of nivolumab and ipilimumab compared to chemotherapy, regardless of PD-L1 status [55]. Subgroup analysis revealed that ever smokers derived a significant survival benefit with the immunotherapy combination (HR 0.72, 95% CI 0.62–0.84), while never smokers did not (HR 0.96, 95% CI 0.66–1.41). In CheckMate9LA, the addition of 2 cycles of chemotherapy to nivolumab and ipilimumab significantly improved survival compared to chemotherapy, again regardless of PD-L1 status [80]. Survival was significantly improved in ever smokers who received chemoimmunotherapy rather than chemotherapy alone (HR 0.62, 95% CI 0.50–0.75). Never smokers appeared to do better with chemotherapy alone (median OS 17.8 months compared to 10.4 months with immunotherapy; HR 1.14, 95% CI 0.66–1.97).

Initial results from the POSEIDON study also showed improved survival for smokers who received dual immunotherapy in combination with chemotherapy, compared to chemotherapy alone, and no significant benefit for never smokers with the checkpoint inhibitor (HR 0.54 current smokers, HR 0.75 former smokers, HR 1.15 never smokers) [81].

In the randomized phase II CCTG BR34 trial, patients received durvalumab plus tremelimumab with or without platinum doublet chemotherapy [82]. Exploratory analyses suggested benefit in never and current smokers with the addition of chemotherapy (HR 0.72, 0.61, respectively). However, former smokers appeared to have similar outcomes whether chemotherapy was added or not (HR 0.91). Patients with low plasma TMB, more commonly female never smokers, were most likely to benefit from the addition of chemotherapy to checkpoint inhibition. This hypothesis generating finding suggests that patients with greater tobacco exposure and higher TMB levels may derive greater benefit from immunotherapy.

### 3.3. Anti-PD(L)1 Therapy in Non-Metastatic NSCLC

#### 3.3.1. Locally Advanced NSCLC–Anti-PD-(L)1 Consolidation Therapy

The PACIFIC trial demonstrated durable survival benefit for patients with unresectable stage III NSCLC with the addition of 12 months of consolidation therapy with durvalumab after concurrent chemoradiation [83]. An unplanned subgroup analysis requested by the European Medicines Agency revealed that patients with PD-L1 negative tumors (<1% tumor expression) did not derive survival benefit compared to placebo. However, both smokers and never smokers appeared to benefit in other sub-analyses (OS HR 0.73, 95% CI 0.59–0.91 in smokers; OS HR 0.42, 95% CI 0.21–0.82 in never smokers), however the numbers of never smokers in the trial was small. Additionally, the potential confounding effects of smoking status, PD-L1 expression and genomic alterations in EGFR and ALK has not been assessed [83].

#### 3.3.2. Perioperative Anti-PD-(L)1 Therapy

IMpower010 was the first randomized phase 3 study to demonstrate improved disease-free survival with 12 months of adjuvant atezolizumab after adjuvant chemotherapy in resected early-stage NSCLC [84]. Subgroup analysis of patients that were former smokers with resected stage II-IIIA PD-L1 positive resected disease revealed that former smokers derived a significantly greater DFS benefit with the addition of atezolizumab (HR 0.82, 95% CI 0.47–0.81). Benefit in the small number of current and never smokers was not seen, with a DFS HR 1.01 and 1.13, respectively. PEARLS/KEYNOTE-091 similarly randomized patients with resected early-stage NSCLC to receive 12 months of pembrolizumab or placebo and also demonstrated improved DFS [85]. Conversely to IMpower010, exploratory analysis found that current smokers derived greatest benefit (HR 0.42, 95% CI 0.23–0.77), compared to former (HR 0.84, 95% CI 0.68–1.04) and never smokers (HR 0.72, 95% CI 0.47–1.13).

CheckMate-816 demonstrated that preoperative chemo-immunotherapy with three cycles of neoadjuvant nivolumab and chemotherapy compared to chemotherapy alone significantly improved event free survival (31.6 months v 20.8 months, HR 0.63) as well as pathological complete response (24% v 2.2%) [86]. Patients that had any history of smoking were more likely to have a pathological complete response with chemo-immunotherapy than never smokers, 25.6% v 10.5%. However, both groups had significantly improved disease-free survival.

### 3.4. Checkpoint Inhibitors and NSCLC with Oncogene Driven Tumors

A growing number of oncogenic drivers are being identified in NSCLC, most commonly in never smokers, although specific alterations may be more common in smokers, such as *KRAS*^G12C^ or *MET.* The role of checkpoint inhibitors for these patients is controversial, as many of the large landmark studies excluded patients with *EGFR* and *ALK* alterations from enrolment. Retrospective analyses in the metastatic setting have demonstrated that response rates are lower with anti-PD-(L)1 monotherapy in patients with oncogenic drivers in tumor [87,88]. While many patients with molecular driver mutations in their tumor may not derive significant benefit from checkpoint inhibitors, there are exceptions [89]. Some retrospective studies have found that amongst driver mutation tumors, particularly EGFR, smokers were again more likely than never smokers to respond to checkpoint inhibitor therapy [90,91]. The identification of patients with oncogenic driver who are more likely to respond, and the optimal sequencing and combination of targeted therapies, chemotherapy and immunotherapy remains unclear and continues to be an ongoing area of interest and active research. These intricacies are beyond the scope of this review and we refer readers to an excellent chapter discussing such issues in more depth [92].

### 3.5. Checkpoint Inhibitors and Small Cell Lung Cancer

Small cell lung cancer (SCLC) accounts for 15% of new lung cancer diagnoses, and is strongly associated with cigarette smoking. It is an aggressive malignancy with poor long-term outcomes. Small cell lung cancer has a high mutational load and is thought to be a potential immunogenic tumor type, due to its association with autoimmune paraneoplastic syndromes. Despite the potential immunogenicity, the benefit of checkpoint inhibitors in small cell lung cancer is more modest compared to that seen in NSCLC. Studies of single-agent ICIs in previously treated SCLC failed to demonstrate survival benefit [93,94]. However, the addition of checkpoint inhibitors to chemotherapy in the first-line setting has demonstrated improved survival compared to chemotherapy alone [95,96,97]. Despite having a high TMB, the tumor immune microenvironment in small cell is thought to be more immunosuppressive, with low levels of CD8^+^, high levels of Tregs and other suppressive immune cells [98,99] which may contribute to the modest benefits seen to date. This suggests that the biology of SCLC and how it responds to ICIs may be different than that seen with NSCLC.

## 4. Future Directions to Understanding Tobacco Use and ICI outcomes

Our review has identified that patients with a history of tobacco use may derive a greater benefit from ICI therapy than patients without a smoking history. This association was seen in both ICI monotherapy, with pre-treated and treatment naïve patients, and when used in combination with cytotoxic chemotherapy.

Our results are supported by other studies. A recent meta-analysis by Dai et al. [100] found that ICI monotherapy and dual ICIs improved survival in ever smokers but not never smokers, regardless of PD-L1, and that the addition of chemotherapy was able to improve survival for both groups. Several real-world studies and analyses further support these observed associations [101,102,103].

Further studies comparing ICI to chemotherapy in trials across multiple malignancies also found that survival was improved in smokers but was not significantly different in never-smokers with NSCLC, head and neck and urothelial cancers [104,105]. ICIs are being increasingly used in these disease sites, as well as other malignancies where the association between disease site and tobacco use is less strong. Understanding how tobacco exposure can impact their responses will be important to guide treatment decisions between systemic therapy options within each disease site. This is likely to be unique to each disease site as prior studies have demonstrated that tobacco exposure can have differential effects on the immune micro-environment based on tumor subtype, which in turn is correlated with the clinical response to treatment [52].

In a recent systematic scoping review and meta-analyses on cancer cooperative group clinical trials by our group [106], we identified that only 25% of trials run by cancer cooperative groups collect baseline tobacco information, predominantly those involving lung or head and neck cancers. Among the trials that collected information on baseline tobacco data, only half reported or presented any analysis of the impact of tobacco use on clinical trial outcomes. Furthermore, none of these identified clinical trials reported on or evaluated the impact of changes in tobacco use on treatment outcomes after study enrolment. This is a missed opportunity to better understand how tobacco use can impact outcomes for patients on systemic therapy, including ICIs. We recommend that clinical trials evaluating ICIs and other systemic therapies start collecting baseline and follow-up tobacco history information, particularly in disease sites not traditionally associated with tobacco use. Implementation of tools to routinely assess tobacco use in trials is feasible in clinical trials [107,108] and will allow for secondary analysis of clinical trial data to assess how tobacco exposure can impact ICI outcomes.

In addition to routine collection of tobacco use in clinical trials, standardized documentation of tobacco status may be beneficial. As seen in our current review and also in our group’s prior scoping review, there is significant heterogeneity in the reporting and analysis of tobacco status in clinical trials with some studies reporting ever/never smoking status, while other studies further separate patients into current/ex-smokers/never smoker status and not all studies routinely report smoking intensity (i.e., pack years). This does impact our understanding of how tobacco can affect the response to immune checkpoint inhibitors and other systemic therapy options. The use of e-cigarettes and cannabis productions, and their role in lung cancer is even less understood, but with rising use, this data will also be important to document in clinical settings.

From our review, patients with a smoking history tended to have greater response to ICIs. In some first-line trials, differences were observed in the magnitude of benefit seen between patients smoking upon trial enrolment and patients who were ex-smokers at the time of enrolment. Further sub-classifying ever smokers into ex-smokers and current smokers and comparing outcomes using this categorization will help us understand if differences observed are due to any history of tobacco use or are those due to current tobacco use at time of study enrolment. As seen in some of the current lung ICI trials, ex-smokers at the time of study enrolment benefitted more from immunotherapy while current smokers may not have received the same magnitude of benefit. This suggests that any historical tobacco use may be associated with improved outcomes with immunotherapy treatment, while current or continued tobacco use during immunotherapy may lead to poorer outcomes, potentially due to ongoing mechanisms of immunosuppression and evasion. Data presented in our review supports the importance of smoking cessation, especially in these patients planned to commence immunotherapy.

One additional area that has not been well studied to date is the impact of tobacco exposure on toxicities due to immune checkpoint inhibitors. Among previous studies focusing on cytotoxic chemotherapy and targeted therapy options, it has been demonstrated that tobacco can impact treatment response and toxicities through both pharmacodynamic and pharmacokinetic changes in drug metabolism, which, in turn, leads to changes in the clinical response to treatment and potential treatment toxicities [109,110,111,112]. However, less is known about how tobacco can impact toxicities related to immunotherapy. Tobacco exposure can have pro-inflammatory or anti-inflammatory responses on the tumor microenvironment, this may in turn potentially impact risk for immune-related adverse events (i.e., pneumonitis, colitis) differently for each disease site and this should be further investigated in both clinical trials as well as in real-world studies.

Based on our current review, it is evident that outcomes for patients with NSCLC treated with ICIs are affected by their tobacco smoking history. Tobacco is being used in clinical settings to guide treatment decision making, but it needs to be more formally studied. Looking forward, we recommend that future prospective studies evaluating ICIs in NSCLC and other disease sites implement dedicated analyses to test the association between tobacco use and ICI outcomes.
